# Striatal Injury with 6-OHDA Transiently Increases Cerebrospinal GFAP and S100B

**DOI:** 10.1155/2015/387028

**Published:** 2015-05-18

**Authors:** Cristiane Batassini, Núbia Broetto, Lucas Silva Tortorelli, Milene Borsoi, Caroline Zanotto, Fabiana Galland, Tadeu Mello Souza, Marina Concli Leite, Carlos-Alberto Gonçalves

**Affiliations:** ^1^Biochemistry Postgraduate Program, Federal University of Rio Grande do Sul (UFRGS), 90035-003 Porto Alegre, RS, Brazil; ^2^Neuroscience Postgraduate Program, Federal University of Rio Grande do Sul (UFRGS), 90050-170 Porto Alegre, RS, Brazil

## Abstract

Both glial fibrillary acidic protein (GFAP) and S100B have been used as markers of astroglial plasticity, particularly in brain injury; however, they do not necessarily change in the same time frame or direction. Herein, we induced a Parkinson's disease (PD) model via a 6-OHDA intrastriatal injection in rats and investigated the changes in GFAP and S100B using ELISA in the substantia nigra (SN), striatum, and cerebrospinal fluid on the 1st, 7th, and 21st days following the injection. The model was validated using measurements of rotational behaviour induced by methylphenidate and tyrosine hydroxylase in the dopaminergic pathway. To our knowledge, this is the first measurement of cerebrospinal fluid S100B and GFAP in the 6-OHDA model of PD. Gliosis (based on a GFAP increase) was identified in the striatum, but not in the SN. We identified a transitory increment of cerebrospinal fluid S100B and GFAP on the 1st and 7th days, respectively. This initial change in cerebrospinal fluid S100B was apparently related to the mechanical lesion. However, the 6-OHDA-induced S100B secretion was confirmed in astrocyte cultures. Current data reinforce the idea that glial changes precede neuronal damage in PD; however, these findings also indicate that caution is necessary regarding the interpretation of data in this PD model.

## 1. Introduction

Parkinson's disease (PD) is a very common, progressive, and socially important neurodegenerative disorder. It is caused by loss of catecholaminergic dopamine-producing neurons in the* substantia nigra* (SN)* pars compacta* and the consequent reduction in striatal dopamine levels [1]. The intrastriatal injection of 6-hydroxydopamine (6-OHDA) is widely used to investigate PD pathogenesis and mechanisms of neuronal death and to evaluate therapeutic strategies for PD because it induces the loss of the dopaminergic nigrostriatal pathway [2].

In addition to the primary characteristic loss of neurons, PD also involves astrocyte dysfunction [3]. Glial cells are the targets of therapeutic interventions in many neurodegenerative disorders, including PD [4–6]. Protein and mRNA expression of glial fibrillary acidic protein (GFAP), the most used astrocyte marker for injury, supports astroglial involvement in PD and experimental models induced by 6-OHDA [7, 8]. More recently, it has been proposed that dopaminergic neuronal death could be a result of the production/release of neurotoxins from astrocytes, such as glutamate, S100B, cytokines, and reactive oxygen and nitrogen species (e.g., [9–11]).

Both GFAP and S100B have been used as astrocyte markers of brain injury; however, they do not necessarily change in the same time or direction. A better understanding of the glial involvement in dopaminergic degeneration model will allow us to improve our data interpretation regarding PD. Thus, our aim was to investigate the changes in GFAP and S100B in the SN, striatum, and cerebrospinal fluid (CSF) on the 1st, 7th, and 21st days following the striatal injection of 6-OHDA in rats. We observed that glial changes precede neuronal damage, based on the content of tyrosine-hydroxylase (TH), a marker for dopaminergic neurons. Moreover, interestingly, the initial change in CSF S100B was apparently more related to the mechanical lesion of the model than 6-OHDA toxicity.

## 2. Methods

### 2.1. Animals

Wistar rats were obtained from our breeding colony (Department of Biochemistry, Federal University of Rio Grande do Sul, Brazil) and were maintained under controlled light and environmental conditions (12 h light/12 dark cycle at a constant temperature of 22 ± 1°C) with free access to food and water.

All animal experiments were performed in accordance with the National Institutes of Health Guide for the Care and Use of Laboratory Animals (NIH Publications no. 80-23) and followed the regulations of the local animal house authorities (UFRGS, no. 23422).

### 2.2. Surgery Procedures

To analyse the* in vivo* effect of the toxin 6-OHDA, seventy-two adult male rats (300–380 g, 110 days old) were divided into two groups: sham/vehicle (*n* = 36) and 6-OHDA (*n* = 36) groups. Behaviour and biochemical analyses were performed on day 1 (*n* = 12 per group), day 7 (*n* = 12), or day 21 (*n* = 12) after surgery. Half of the animals of each group, in each time point, was used for behaviour analysis and immunohistochemistry, and the other half was used for biochemical analysis (after CSF collecting and brain dissection) to evaluate the contents of S100B, GFAP, and TH.

The rats were anesthetised with equithesin (3 mL/kg; i.p.; 2.12 g of chloral hydrate, 0.48 g of pentobarbital, 1.08 g of MgSO_4_, 21.4 mL of propylene glycol, and 5.74 mL ethanol and water, q.s.p. 50 mL) and placed in a stereotaxic apparatus. The animals subsequently received three injections of vehicle (sham-group; 0.2% ascorbic acid/0.9% NaCl saline solution; 6 *μ*L) or 6-OHDA (21 *μ*g, 6 *μ*L; Sigma, 162957, USA) into the right striatum over 20 min. For each injection, 2 *μ*L was infused, 0.5 *μ*L/min, according to the following coordinates: AP: 0 mm, LL: −2.8 mm, and DV: 0 mm from the Bregma; AP: −0.5 mm, LL: −3.9 mm, and DV: −6.0; and AP: −1.2 mm, LL: −4.1 mm, and DV: −6 mm; incisor bar: 0 [12]. An injection needle attached to a 10 *μ*L microsyringe (Hamilton, 701N, USA) and an infusion pump (Insight, Brazil) was used. The needle was kept in the brain for 4 min after each infusion to allow drug diffusion. The rats received postoperative care until they were awake and were returned to their home cages.

### 2.3. Rotational Activity Analysis

One, seven, or 21 days after the surgical procedure, the animals were challenged with 40 mg/kg of methylphenidate i.p. (Ritalina, Novartis, Brazil) [13] and immediately placed in an 80 cm diameter circular arena for 30 min. The numbers of rotations were recorded.

### 2.4. Immunohistochemistry

Immunohistochemistry was performed according to Brolese and coworkers [14]. The rats were anesthetised as previously described and perfused through the left cardiac ventricle using a peristaltic pump (Milan, Brazil) with 0.9% NaCl solution followed by 4% paraformaldehyde in phosphate buffer saline (PBS), pH 7.4. The brains were removed and left for postfixation in the same fixative solution at 4°C for 24 h. The material was subsequently cryoprotected by immersing the brain in 15 and 30% sucrose in PBS at 4°C. Then, the brains were frozen by immersion in isopentane cooled in liquid nitrogen and stored in a freezer (−80°C) for subsequent analyses. The brains were sectioned in coronal plane (45 *μ*m) on a cryostat (Leica). The free-floating sections that contained the striatum (between 0 and −1 mm from Bregma [15]) or SN (between −5 and −6 mm from bregma [15]) were then incubated with polyclonal anti-GFAP from rabbit (Dako, Z0334, Denmark) or anti-TH from rabbit (Millipore, AB152, Germany), respectively, diluted 1 : 3000 in 0.4% PBS-Triton X-100 and 2% bovine serum albumin (BSA), for 48 h at 4°C. After washing several times with PBS, the tissue sections were incubated with Alexa Fluor 488 (goat anti-rabbit-IgG; green fluorescence; Invitrogen, A11008) at room temperature for 1 h. Alexa Fluor secondary antibody was diluted 1 : 500 in 0.4% PBS-Triton X-100 and 2% BSA. The sections were then washed several times, mounted on slides with FluorSave (Calbiochem), and covered with coverslips. Images were viewed with a Nikon microscope and transferred to a computer with a digital camera.

### 2.5. Cerebrospinal Fluid, Blood, and Brain Samples

In the second set after surgery, the animals were anesthetised as previously described and then positioned in a stereotaxic holder; the CSF was obtained by cisterna magna puncture. The animals were subsequently sacrificed by decapitation. The brains were removed, placed in cold PBS, and the striatum and ventral midbrain area, which contained the SN, were removed by free-hand dissection. SN was dissected as described by Ding and coworkers [16]. Briefly, a 0.8–1.0 mm thick coronal section of the mesencephalon was made using a scalpel and regions containing SN* pars compacta* were isolated. After being removed, striatum was sliced (0.3 mm) using a Mcllwain tissue chopper. SN was not sliced, due to the reduced size. The SN, striatum slices, and CSF were frozen (−80°C) until further analysis.

### 2.6. Western Blotting

The brain samples were homogenised and equal amounts (20 *μ*g) of proteins from each sample were boiled in sample buffer (0.0625 M Tris-HCl, pH 6.8, 2% (w/v) SDS, 5% (w/v) b-mercaptoethanol, 10% (v/v) glycerol, and 0.002% (w/v) bromophenol blue) and electrophoresed in 10% (w/v) SDS-polyacrylamide gel. The proteins were blotted onto a nitrocellulose membrane [17]. The monoclonal antibody anti-TH (Sigma, T1299, USA) was used at a dilution of 1 : 1000. Following incubation with the primary antibody overnight at 4°C, the membranes were washed and incubated with peroxidase-conjugated anti-mouse immunoglobulin (IgG) at a dilution of 1 : 2000. The chemiluminescent reactions were developed using luminol as the substrate (ECL Western Blotting Analysis System, GE Healthcare, Brazil) and registered on radiographic film. The immunocontent of TH was determined as a ratio of the optical density (OD) of the protein band (TH)/OD of the *β*-actin band. The bands were quantified using Scion Image software [14].

### 2.7. Cell Cultures

Primary astrocyte cultures were prepared from eight Wistar rats (1-2 days old) to evaluate 6-OHDA* in vitro* effect on secretion and intracellular content of S100B.

Astrocyte cultures were prepared as previously described [18]. Fetal calf serum (FCS), Dulbecco's modified Eagle's medium (DMEM), and other materials for cell culture were purchased from Gibco (USA). Briefly, the cerebral cortices of new-born Wistar rats were removed and mechanically dissociated in Ca^2+^- and Mg^2+^-free balanced salt solution, pH 7.4, which contained (in mM): 137 NaCl, 5.36 KCl, 0.27 Na_2_HPO_4_, 1.1 KH_2_PO_4_, and 6.1 glucose. The cortices were cleaned of meninges and mechanically dissociated via sequential passage through a Pasteur pipette. After centrifugation at 1400 RPM for 5 min, the pellet was resuspended in DMEM (pH 7.6) supplemented with 8.39 mM HEPES, 23.8 mM NaHCO_3_, 0.1% amphotericin, 0.032% gentamicin, and 10% FCS. The cultures were maintained in DMEM that contained 10% FCS in 5% CO_2_/95% air at 37°C, allowed to grow to confluence, and used at 15 days* in vitro*. The medium was replaced by DMEM with 1% FCS in the presence of 6-OHDA (200 *μ*M) or vehicle for 24 h. A GSH saline solution was used as the vehicle [19].

### 2.8. S100B Measurement

S100B was measured by ELISA as previously described [20]. Briefly, 50 *μ*L of sample plus 50 *μ*L of Tris buffer was incubated for 2 h on a microtiter plate previously coated with monoclonal anti-S100B antibody (Sigma, S2532, USA). Polyclonal anti-S100 (Dako, Z0311, Denmark) was incubated for 30 min; peroxidase-conjugated anti-rabbit antibody (GE, NA934V, UK) was subsequently added for an additional 30 min. The colour reaction with *ο*-phenylenediamine (Sigma, P8936, USA) was measured at 492 nm. The standard S100B curve ranged from 0.002 to 1 ng/mL.

### 2.9. GFAP Measurement

ELISA for GFAP was performed as previously described [21] by coating microtiter plates with 100 *μ*L samples for 24 h at 4°C. Incubation with a polyclonal anti-GFAP from rabbit (Dako, Z0334, Denmark) for 1 h was followed by incubation with a secondary antibody conjugated with peroxidase for 1 h at room temperature (GE, NA934V, UK). A colorimetric reaction with *ο*-phenylenediamine (Sigma, P8936, USA) was measured at 492 nm. The standard human GFAP (from Calbiochem) curve ranged from 0.1 to 5 ng/mL.

### 2.10. Protein Determination

The protein content was measured by Lowry's method using BSA as the standard [22].

### 2.11. Statistical Analysis

Data are reported as the mean ± standard error and were analysed by Student's *t*-test (when two groups were considered) or two-way analysis of variance (ANOVA) followed by Tukey's test, which assumed *P* < 0.05 as significant.

## 3. Results

### 3.1. Behavioural and Tyrosine-Hydroxylase Content Changes 1, 7, and 21 Days after 6-OHDA Striatal Injection

**Figure 1:**
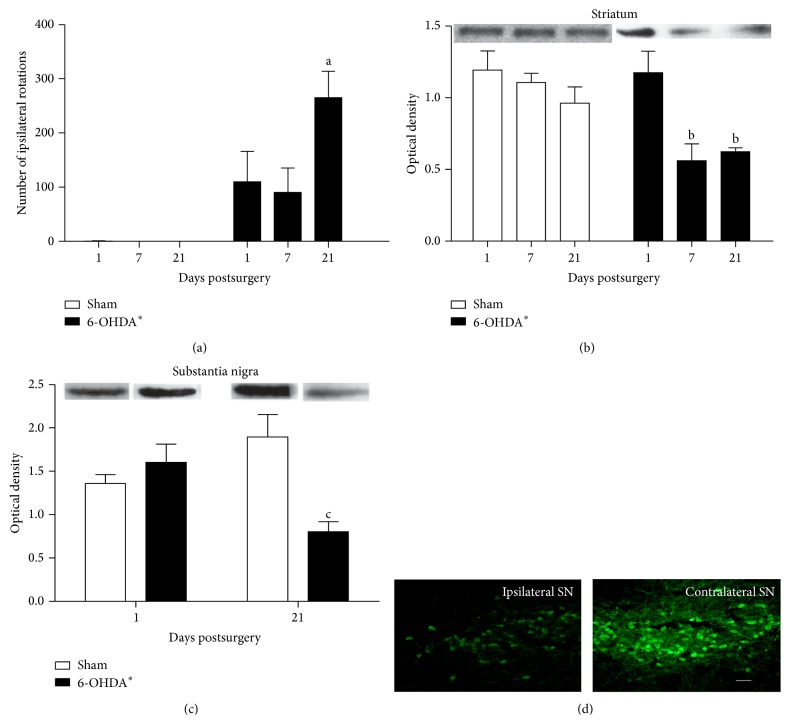
Rotational behaviour and tyrosine-hydroxylase immunocontent in rats administered an intrastriatal 6-OHDA injection. (a) the number of ipsilateral rotations after a methylphenidate challenge. Tyrosine-hydroxylase immunocontent in the striatum (b) and substantia nigra (c) at 1st, 7th, or 21st days after 6-OHDA intrastriatal administration. The striatum and substantia nigra were dissected and homogenized for the measurement of the tyrosine-hydroxylase by Western Blotting analysis. Each value represents the mean ± standard error from at least 4 rats per group. ^∗^The 6-OHDA-group is significantly different than the sham/vehicle-group (two-way ANOVA, *P* < 0.05), in (a), (b), and (c). ^a^Different from 6-OHDA-lesioned rats at days 1 and 7; ^b^different from 6-OHDA-group at 1 day; ^c^different from 6-OHDA-group 1 day after and different, from sham-group 21 days after surgery (two-way ANOVA followed by Tukey's post hoc test, *P* < 0.05). Panel (d) shows a representative TH-immunohistochemistry for dopamine-producing cells in the ipsilateral and contralateral sides of the substantia nigra (SN)* pars compacta* in a 6-OHDA-lesioned rat 21 days after surgery. Scale bar = 10 *μ*m.

To validate the 6-OHDA rat model, we performed a classic behavioural test. An intraperitoneal injection of methylphenidate (40 mg/kg; Ritalina) induced ipsilateral rotational behaviour in 6-OHDA-lesioned rats as shown in Figure 1(a) (significant main effect of group, two-way ANOVA, *F*(1,11) = 32.65, *P* < 0.001). There was also a time effect (two-way ANOVA, *F*(2,11) = 4.86, *P* = 0.031), which was indicated by the increased number of rotations in the 6-OHDA-lesioned rats at day 21 after the lesion compared with days 1 (Tukey's post hoc test, *P* = 0.024) and 7 (Tukey's post hoc test, *P* = 0.006).

In addition, the striatal slices (Figure 1(b)) and ventral midbrain area that contained the SN (Figure 1(c)) from rats 1, 7, or 21 days post-6-OHDA were analysed by Western Blotting for TH. The TH immunocontent in the striatum was different between the 6-OHDA-group and the sham-group (two-way ANOVA, *F*(1,17) = 11.19, *P* = 0.004). There was a time effect (two-way ANOVA, *F*(2,17) = 7.74, *P* = 0.004) in 6-OHDA-group, in which the 1 day group had more TH immunocontent than the 7 (Tukey's post hoc test, *P* = 0.002) and 21 (Tukey's post hoc test, *P* = 0.006) day groups.

Similarly, in the SN, the 6-OHDA-group was significantly different from the sham-group (two-way ANOVA, *F*(1,16) = 5.34, *P* = 0.034); a reduction in the TH immunocontent in the 6-OHDA-group 21 days after surgery was identified compared with the 6-OHDA-group 1 day after and the sham-group 21 days after surgery (Figure 1(c), Tukey's post hoc test, *P* = 0.008 and *P* < 0.001, resp.). Representative TH-immunohistochemistry of the SN is shown in Figure 1(d). The TH-containing cells were reduced in the ipsilateral SN* pars compacta* in the 6-OHDA-group 21 days after surgery.

### 3.2. GFAP and S100B Content in the Striatum and SN of Rats Administered a 6-OHDA Striatal Injection

**Figure 2:**
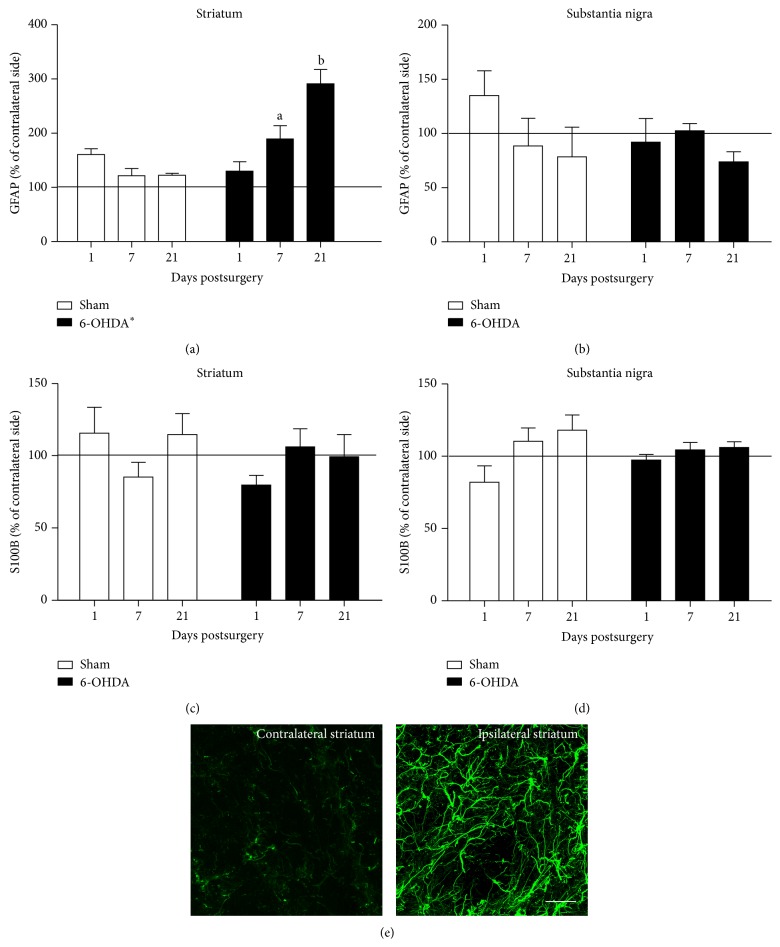
GFAP and S100B content in the striatum and substantia nigra of rats administered an intrastriatal 6-OHDA injection. Brain samples were analysed at 1, 7, or 21 days after the infusion of 6-OHDA, and the GFAP ((a), (b)) and S100B ((c), (d)) contents were measured by ELISA. Each value represents the mean ± standard error, which was expressed as the percentage of the contralateral unlesioned side (assumed to be 100%, indicated by line) from at least 5 rats per group. ^∗^Different from sham-group (two-way ANOVA, *P* < 0.001). ^a^Different from sham at 7 days; ^b^different from sham at 21 days, and different from 6-OHDA at 1 and 7 days (two-way ANOVA followed by Tukey's post hoc test, *P* < 0.05). (e) Immunohistochemistry for GFAP in the striatum at 21 days after surgery. Representative photomicrographs of a 6-OHDA-lesioned rat show the striatum ipsilateral to the 6-OHDA infusion on the right and the striatum contralateral to the infusion on the left. Scale bar = 25 *μ*m.

The immunocontents of GFAP and S100B were analysed using ELISA in the striatum slices and ventral midbrain area that contained the SN. The values are expressed as the percentage of the contralateral side that did not undergo stereotaxic surgery (assumed to be 100%).  Figure 2(a) shows the GFAP changes in the striatum. The striatal GFAP content was increased in the 6-OHDA group of rats (two-way ANOVA, *F*(1,38) = 18.80, *P* < 0.001). There was also an effect of time (two-way ANOVA, *F*(2,38) = 5.61, *P* = 0.007), in which the 6-OHDA group of rats exhibited increased levels of striatal GFAP content 7 and 21 days after surgery compared with the sham-group at the same times (Tukey's post hoc test, *P* = 0.01 and *P* < 0.001, resp.); furthermore, the GFAP content was higher 21 days after the toxin injection (Tukey's post hoc test, *P* < 0.001 and *P* = 0.001 compared with 1 and 7 days, resp.). To confirm the changes in GFAP identified in the rats injected with 6-OHDA, we conducted a GFAP immunohistochemistry study in striatum tissue on 7 (data not shown) and 21 days (Figure 2(e)). However, no changes in the GFAP content were identified in the SN (Figure 2(b)) (two-way ANOVA, *P* > 0.05).

In contrast to GFAP, no changes were identified in the S100B content in the striatum (Figure 2(c)) (two-way ANOVA, *P* > 0.05) or the SN (Figure 2(d)) (two-way ANOVA, *P* > 0.05).

### 3.3. GFAP and S100B Levels in the CSF of Rats Administered a 6-OHDA Injection

**Figure 3:**
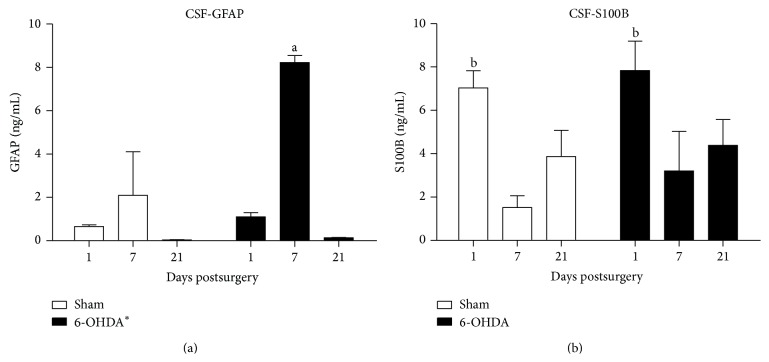
GFAP and S100B levels in the cerebrospinal fluid (CSF) of rats administered a 6-OHDA injection. CSF was obtained by cisterna magna puncture, and GFAP (a) and S100B (b) were measured by ELISA. Each value represents the mean ± standard error from at least 4 rats per group. ^∗^Different from sham-group (two-way ANOVA, *P* = 0.002). ^a^Different from sham at 7 days and different from 6-OHDA at 1 and 21 days after surgery; ^b^different from S100B content at days 7 and 21 (two-way ANOVA followed by Tukey's post hoc test, *P* < 0.05).

In addition to the tissue changes of astrocyte markers, we evaluated their content in the CSF. The CSF content of GFAP, which was evaluated by ELISA, was not altered in the sham-group at 1 to 21 days after stereotaxic surgery (Figure 3(a)). However, in the 6-OHDA group, there was a significant increase (two-away ANOVA, *F*(1,9) = 18.33, *P* = 0.002). There was a time effect (two-way ANOVA, *F*(2,9) = 36.37, *P* < 0.001); that is, we observed an increase on the 7th day after surgery; however, this change was transient because it was not observed on the 21st day (Tukey's post hoc test, *P* < 0.001).

The CSF S100B content is shown in Figure 3(b). There was a time effect (two-way ANOVA, *F*(2,33) = 10.415, *P* < 0.001) in the 6-OHDA and sham-groups. We observed a clear increment in both groups on 1 day after stereotaxic surgery (Tukey's post hoc test, *P* < 0.001 and *P* = 0.026, resp., versus 7 and versus 21 days). No significant changes were identified 7 or 21 days after surgery in the 6-OHDA and sham-groups.

### 3.4. S100B Secretion in Cultured Astrocytes Exposed to 6-OHDA

**Figure 4:**
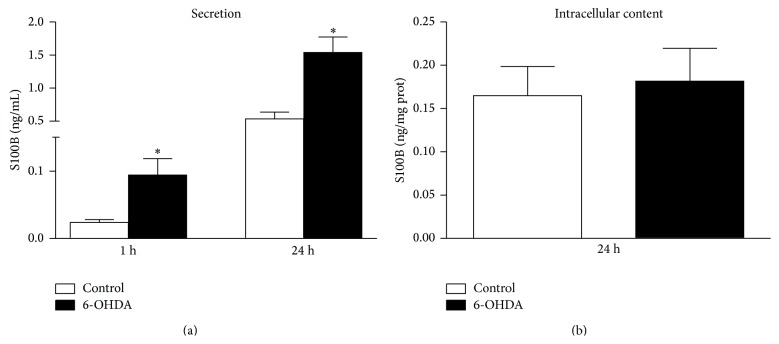
S100B secretion and S100B content in astrocyte cultures from rats exposed to 6-OHDA 200 *μ*M. After confluence, the medium was replaced by DMEM 1% FCS in the presence or absence of 6-OHDA 200 *μ*M. S100B secretion was measured by ELISA at 1 and 24 h (a). The intracellular content of S100B (b) was measured by ELISA at 24 h. Each value represents the mean ± standard error of at least 8 independent experiments performed in triplicate. ^∗^Different from the control (Student's *t*-test, *P* < 0.05).

Considering that the mechanical injury (by the needle) in this model caused an increase in CSF S100B, it was not possible to determine if 6-OHDA* per se* induced S100B secretion. Therefore, we exposed astrocyte cultures to 6-OHDA and evaluated S100B secretion. The 6-OHDA induced an increase in S100B secretion at 1 and 24 h (Figure 4(a)) (Student's *t*-test, *P* = 0.009 and *P* = 0.005, resp.), without affecting the intracellular content of S100B during this time (Figure 4(b)) (Student's *t*-test, *P* > 0.05).

## 4. Discussion

6-OHDA-induced PD in rats has been a useful model to evaluate potential therapeutic treatment strategies [2]. The protocol used induced, as expected, a slow, progressive, and partial lesion of the nigrostriatal pathway in a retrograde manner over a period of up to 3 weeks [23]. This lesion was confirmed by rotational behaviour induced by methylphenidate, as well as by a decrease in TH immunoreactivity in the striatum and SN 21 days after the striatal lesion.

The hallmark of PD is the loss of dopaminergic neurons in the nigrostriatal pathway, which is accompanied by gliosis [24] and commonly measured by an increment of GFAP immunoreactivity. The astroglial response appears to be essential to understand and propose therapeutic strategies for PD and other neurodegenerative disorders (e.g., [6]) and to potentially understand PD pathogenesis [9].

Gliosis based on GFAP increment occurs following the intrastriatal administration of 6-OHDA [7]. We identified an increment of GFAP in the ipsilateral striatum at 7 and 21 days after 6-OHDA administration, which is consistent with previous findings [8, 25]. Despite the TH decrease in the SN on the 21st day, no significant gliosis was observed in the SN or prior to this point.

To our knowledge, this is the first measurement of CSF GFAP (and S100B) in the 6-OHDA model of PD. CSF GFAP increments have been clinically identified in some conditions of acute brain injury, such as stroke, as well as chronic conditions, such as Alzheimer's disease or vascular dementia. However, no changes were identified in PD patients in two previous studies [26, 27]. Here, we identified a transitory increment of CSF GFAP on the 7th day after the lesion induced by 6-OHDA, which may reflect the acute scenario of damage. This finding allows us to speculate that it would be possible to detect the increment of CSF GFAP in the beginning of the disease or during periods of exacerbation.

In two investigations with PD patients did not identify changes in CSF S100B [27, 28]; however, a more recent study identified an increase [29]. In addition, this previous study identified S100B-gliosis in the SN in postmortem patients. S100B has been used as a general marker of brain damage [30, 31]; however, some authors have suggested an active role of this protein in the pathogenesis of PD [9, 29, 32]. Conflicting results are also observed in a PD model with intrastriatal administration of 6-OHDA. For example, an increment of S100B positive astrocytes has been described in the striatum, SN, and contralateral nuclei [33]. However, in a previous study, despite a GFAP increment, no changes were identified in striatal S100B immunoreactivity from 2 to 14 days after 6-OHDA administration [34]. Here, we did not identify changes in S100B in the striatum or SN when the S100B content was measured by ELISA. However, we identified CSF S100B increment (about 7 ng/mL) 1 day after stereotaxic surgery. This increment is clear, based on our previous measurements in basal conditions (<2 ng/mL) [20, 31].

This increase in S100B at 1 day was observed in the 6-OHDA and sham-groups. This increment is likely due to the mechanical lesion induced by the needle. Striatal astroglial activation (based on GFAP immunoreactivity) induced by a needle lesion has been described [35]. It is possible to observe an increase in the GFAP striatal levels measured by ELISA (in the sham and 6-OHDA groups) compared with the respective contralateral nuclei (unpaired Student's *t*-test, *P* < 0.05). The increment on CSF S100B reflects astrocyte secretion in response to an acute brain lesion. This secretion is not restricted to the striatum, and the secreted amount is frequently small compared with the intracellular content, which explains why extracellular and intracellular changes are not necessarily associated [30]. To determine if 6-OHDA is able to induce astroglial activation (looking at S100B secretion), we evaluated astrocyte cultures exposed to 6-OHDA. At 200 *μ*M, 6-OHDA was able to induce S100B secretion at 1 and 24 h, without significant changes in the intracellular content.

It is important to note some limitations of this work. First, we chose the 6-OHDA model of PD; however, we are aware that it is necessary to confirm these data regarding glial parameters in other models. Second, a mechanical lesion induced by the needle masked the lesion induced by 6-OHDA. In future studies in this PD model, the measurement of CSF S100B between 2 and 5 days would help to identify a specific increment of CSF S100B induced by 6-OHDA. Third, this study focused on two classical markers of astroglial activation. However, it is important to consider the astroglial heterogeneity and that other astrocyte markers also deserve attention, such as Aquaporin-4 [36] or Glt-1 [37], particularly because astrocyte dysfunction has been demonstrated to be involved in PD initiation and progression.

## 5. Conclusions

In summary, we evaluated two markers of astroglial plasticity in a PD model in rats obtained with intrastriatal administration of 6-OHDA. The current data reinforce several ideas regarding PD; however, the findings also indicate several limitations of the model used for this disorder. Glial activation (signalled by GFAP increment in the striatum and CSF) precedes neuronal damage; however, the initial change in CSF S100B in this PD model was related to a mechanical lesion, which masks 6-OHDA glial toxicity. The 6-OHDA glial toxicity was confirmed by increased S100B secretion in astrocyte cultures.

## References

[1] Beitz J. M. (2014). Parkinson's disease: a review. *Frontiers in Bioscience*.

[2] Blandini F., Armentero M.-T. (2012). Animal models of Parkinson’s disease. *FEBS Journal*.

[3] Halliday G. M., Stevens C. H. (2011). Glia: Initiators and progressors of pathology in Parkinson's disease. *Movement Disorders*.

[4] Cicchetti F., Barker R. A. (2014). The glial response to intracerebrally delivered therapies for neurodegenerative disorders: is this a critical issue?. *Frontiers in Pharmacology*.

[5] Yokoyama H., Uchida H., Kuroiwa H., Kasahara J., Araki T. (2011). Role of glial cells in neurotoxin-induced animal models of Parkinson's disease. *Neurological Sciences*.

[6] Fenoy A. J., Goetz L., Chabardès S., Xia Y. (2014). Deep brain stimulation: are astrocytes a key driver behind the scene?. *CNS Neuroscience and Therapeutics*.

[7] Nakagawa T., Schwartz J. P. (2004). Gene expression profiles of reactive astrocytes in dopamine-depleted striatum. *Brain Pathology*.

[8] Carbone M., Duty S., Rattray M. (2012). Riluzole neuroprotection in a Parkinson's disease model involves suppression of reactive astrocytosis but not GLT-1 regulation. *BMC Neuroscience*.

[9] Niranjan R. (2014). The role of inflammatory and oxidative stress mechanisms in the pathogenesis of Parkinson’s disease: focus on astrocytes. *Molecular Neurobiology*.

[10] Barcia C., Ros C., Annese V. (2011). IFN-*γ* signaling, with the synergistic contribution of TNF-*α*, mediates cell specific microglial and astroglial activation in experimental models of Parkinson's disease. *Cell Death & Disease*.

[11] Mena M. A., De Bernardo S., Casarejos M. J., Canals S., Rodríguez-Martín E. (2002). The role of astroglia on the survival of dopamine neurons. *Molecular Neurobiology*.

[12] Kirik D., Rosenblad C., Björklund A. (1998). Characterization of behavioral and neurodegenerative changes following partial lesions of the nigrostriatal dopamine system induced by intrastriatal 6-hydroxydopamine in the rat. *Experimental Neurology*.

[13] Silvestrin R. B., de Oliveira L. F., Batassini C., Oliveira A., e Souza T. M. (2009). The footfault test as a screening tool in the 6-hydroxydopamine rat model of Parkinson's disease. *Journal of Neuroscience Methods*.

[14] Brolese G., Lunardi P., Broetto N. (2014). Moderate prenatal alcohol exposure alters behavior and neuroglial parameters in adolescent rats. *Behavioural Brain Research*.

[15] Paxinos G., Watson P. (1998). *The Rat Brain in Stereotaxis Coordinates*.

[16] Ding Y. M., Jaumotte J. D., Signore A. P., Zigmond M. J. (2004). Effects of 6-hydroxydopamine on primary cultures of substantia nigra: Specific damage to dopamine neurons and the impact of glial cell line-derived neurotrophic factor. *Journal of Neurochemistry*.

[17] Zanotto C., Abib R. T., Batassini C. (2013). Non-specific inhibitors of aquaporin-4 stimulate S100B secretion in acute hippocampal slices of rats. *Brain Research*.

[18] Gottfried C., Valentim L., Salbego C., Karl J., Wofchuk S. T., Rodnight R. (1999). Regulation of protein phosphorylation in astrocyte cultures by external calcium ions: specific effects on the phosphorylation of glial fibrillary acidic protein (GFAP), vimentin and heat shock protein 27 (HSP27). *Brain Research*.

[19] Soto-Otero R., Méndez-Álvarez E., Hermida-Ameijeiras Á., Muñoz-Patiño A. M., Labandeira-Garcia J. L. (2000). Autoxidation and neurotoxicity of 6-hydroxydopamine in the presence of some antioxidants: potential implication in relation to the pathogenesis of Parkinson's disease. *Journal of Neurochemistry*.

[20] Leite M. C., Galland F., Brolese G. (2008). A simple, sensitive and widely applicable ELISA for S100B: methodological features of the measurement of this glial protein. *Journal of Neuroscience Methods*.

[21] Tramontina F., Leite M. C., Cereser K. (2007). Immunoassay for glial fibrillary acidic protein: antigen recognition is affected by its phosphorylation state. *Journal of Neuroscience Methods*.

[22] Peterson G. L. (1977). A simplification of the protein assay method of Lowry *et al.* which is more generally applicable. *Analytical Biochemistry*.

[23] Przedbroski S., Leviver M., Jiang H. (1995). Dose-dependent lesions of the dopaminergic nigrostriatal pathway induced by instrastriatal injection of 6-hydroxydopamine. *Neuroscience*.

[24] Knott C., Stern G., Wilkin G. P. (2000). Inflammatory regulators in Parkinson's disease: iNOS, lipocortin-1, and cyclooxygenases-1 and -2. *Molecular and Cellular Neuroscience*.

[25] Aponso P. M., Faull R. L. M., Connor B. (2008). Increased progenitor cell proliferation and astrogenesis in the partial progressive 6-hydroxydopamine model of Parkinson's disease. *Neuroscience*.

[26] Holmberg B., Rosengren L., Karlsson J.-E., Johnels B. (1998). Increased cerebrospinal fluid levels of neurofilament protein in progressive supranuclear palsy and multiple-system atrophy compared with Parkinson's disease. *Movement Disorders*.

[27] Abdo W. F., De Jong D., Hendriks J. C. M., Horstink M. W. I. M., Kremer B. P. H., Bloem B. R. (2004). Cerebrospinal fluid analysis differentiates multiple system atrophy from Parkinson's disease. *Movement Disorders*.

[28] Abdo W. F., Bloem B. R., Van Geel W. J., Esselink R. A. J., Verbeek M. M. (2007). CSF neurofilament light chain and tau differentiate multiple system atrophy from Parkinson's disease. *Neurobiology of Aging*.

[29] Sathe K., Maetzler W., Lang J. D. (2012). S100B is increased in Parkinson's disease and ablation protects against MPTP-induced toxicity through the RAGE and TNF-*α* pathway. *Brain*.

[30] Gonçalves C.-A., Concli Leite M., Nardin P. (2008). Biological and methodological features of the measurement of S100B, a putative marker of brain injury. *Clinical Biochemistry*.

[31] Guerra M. C., Tortorelli L. S., Galland F. (2011). Lipopolysaccharide modulates astrocytic S100B secretion: a study in cerebrospinal fluid and astrocyte cultures from rats. *Journal of Neuroinflammation*.

[32] Liu Y., Buck D. C., Neve K. A. (2008). Novel interaction of the dopamine D_2_ receptor and the Ca^2+^ binding protein S100B: role in D_2_ receptor function. *Molecular Pharmacology*.

[33] Gomide V. C., Chadi G. (2005). Glial bFGF and S100 immunoreactivities increase in ascending dopamine pathways following striatal 6-OHDA-induced partial lesion of the nigrostriatal system: a sterological analysis. *International Journal of Neuroscience*.

[34] Gordon M. N., Schreier W. A., Ou X., Holcomb L. A., Morgan D. G. (1997). Exaggerated astrocyte reactivity after nigrostriatal deafferentation in the aged rat. *Journal of Comparative Neurology*.

[35] Gomide V. C., Silveira G. A., Chadi G. (2005). Transient and widespread astroglial activation in the brain after a striatal 6-OHDA-induced partial lesion of the nigrostriatal system. *International Journal of Neuroscience*.

[36] Xiao M., Hu G. (2014). Involvement of aquaporin 4 in astrocyte function and neuropsychiatric disorders. *CNS Neuroscience and Therapeutics*.

[37] Massie A., Goursaud S., Schallier A. (2010). Time-dependent changes in GLT-1 functioning in striatum of hemi-Parkinson rats. *Neurochemistry International*.

